# Cocaine-Induced Kidney, Liver, Lung, and Muscle Injury (C-KLM), Presenting With Foot Drop: A Case Report

**DOI:** 10.7759/cureus.50745

**Published:** 2023-12-18

**Authors:** Ifeoma Kwentoh, Sandhya Daniel, Eric S Atiku

**Affiliations:** 1 Medicine, Columbia University, New York, USA; 2 Internal Medicine, Harlem Hospital Center, New York, USA; 3 Medicine, Harlem Hospital Center, New York, USA; 4 General Surgery, Harlem Hospital Center, New York, USA

**Keywords:** transient neuropraxia, cocaine-induced lung injury, substance induced disorders, cocaine induced rhabdomyolysis, cocaine hepatotoxicity, cocaine levamisole-induced vascuiltis

## Abstract

Cocaine-associated organ injury is a well-known phenomenon that may lead to multi-organ failure. Cocaine-induced kidney, liver, lung, and muscle (C-KLM) involvement is an entity with alarmingly high creatinine phosphokinase (CPK) levels exceeding 100,000 U/L. This may have fatal outcomes. Rhabdomyolysis is one of the many mechanisms of kidney injury induced by cocaine intoxication. Sympathomimetic effects of cocaine contribute to muscle Injury in addition to vasoconstriction resulting in muscle ischemia, as well as liver ischemia (shock liver). Furthermore, increased muscular activity from hyperpyrexia, seizures, and agitation due to cocaine use disorder may contribute to muscle breakdown and worsening nephrotoxicity.

The authors detail a case of a 34-year-old male intravenous drug user who presented with an inability to bear weight or move his left lower extremity due to pain, associated with severe edema of his lower extremities of acute onset. He was subsequently noted with foot drop, oliguria, and high blood pressure following cocaine intoxication. The patient began crashing rather quickly and the intensive care unit was recommended. Labs were noted with overwhelming CPK levels over 100,000 U/L and rising for which urgent hemodialysis was initiated. We detail a catastrophic cocaine-induced multi-organ failure with a positive outcome following a multidisciplinary approach.

## Introduction

Cocaine originated from the Andes Mountain region of South America. It is an alkaloid of the coca leaf and was first discovered in 1860, and has since been made largely available from 1970 [1]. Approximately 1-3% of adults in Western countries have abused cocaine at some point in their lifetime [2]. A recent uptrend in the consumption of cocaine has demanded the need to better understand the mechanisms resulting in its ability to lead to multi-organ involvement and failure. Cocaine can be used intranasally, orally, or via intravenous access. It undergoes hepatic metabolism via the P450 pathway and is mostly excreted in urine [3]. A small amount when excreted in urine is non-metabolized for up to 6 hours. The metabolites may be detected up to 3 days post absorption [4]. Cocaine affects nearly all organs and systems. Multi-organ failure secondary to cocaine use in intravenous drug users may include cocaine-induced hepatotoxicity with elevated aspartate transferase (AST) and alanine transaminase (ALT) likely secondary to enzymes released from muscle breakdown (rhabdomyolysis) and this correlates with the creatine phosphokinase (CPK) levels. Other organs affected by cocaine toxicity include the bowels which undergo ischemia, following vasoconstriction and diminished blood flow. Lung injuries of various spectrum and muscle injury may occur. The cardiovascular system is not left out as the sympathomimetic effects of cocaine can result in fatal arrhythmia and cardiac arrest. There are adulterated versions available with levamisole contamination leading to antineutrophil cytoplasmic antibody (ANCA) associated vasculitis. The endpoint of cocaine toxicity is fatal outcomes and an increase in all-cause mortality.

## Case presentation

A 34-year-old man was hospitalized for evaluation of left foot weakness and inability to bear weight on his foot associated with swelling and body rashes for 1-2 days duration. His inability to bear weight on his left lower extremity was also associated with severe leg edema for one-day duration, and oliguria was noted in the last 12 hours of admission. The patient denied any past medical history and endorsed he was feeling well a day prior to presentation. He denied any drugs or alcohol (EtOH) use, facial asymmetry, dysarthria, headaches, visual disturbances, saddle anesthesia/paresthesia, urinary/bowel incontinence/retention, and also denied any history of the human immunodeficiency virus (HIV), diagnosis and intravenous drug use. He had no prior history of dysuria, fevers, arthralgias, rashes, oral ulcers, or lymphadenopathy in the past. Following more prompting, he admitted having fallen asleep after using bags of cocaine and then woke up, with numbness in his left leg. Mother died from complications of lupus; social history, however, was significant for only crack cocaine use, but no smoking. He denied trauma or injecting his leg with illicit substances.

**Figure 1:**
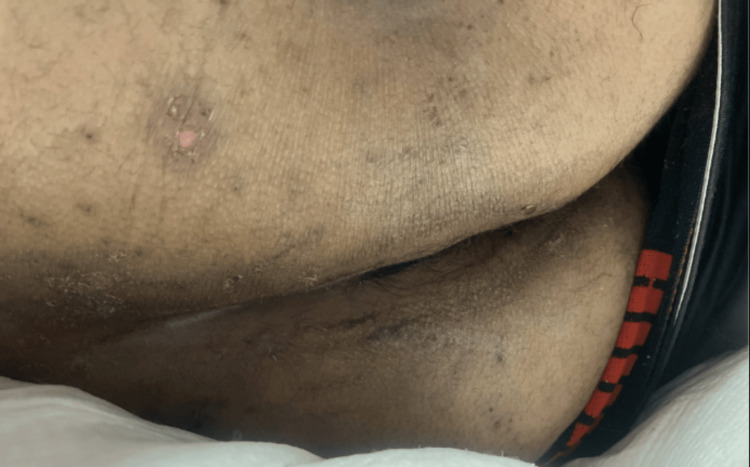
Multiple excoriations and rashes of different healing stages distributed to the skin of the trunk, torso, and buttocks.

Physical examination was significant for hypertension of 179/99 mmHg, other vitals were unremarkable. General examination showed a young, disheveled male in no acute distress. He appeared acutely ill, alert but drowsy, moderately dehydrated, non-icteric, not cyanosed, and afebrile; there were multiple excoriations to bilateral shins, trunk, arms, anterior chest, and buttock area (Figure 1).

A 2x4 cm rectangle-shaped erythema to the left shoulder and erythema to the left medial foot was noted. The left foot was noted with decreased sensation and decreased strength with 2+ dorsalis pedis pulses and 2-second capillary refills. There was 5/5 strength and sensation proximal to the ankle. There was no other strength deficit/sensation deficit in the left lower extremities. There were diffuse rales on chest examination. He had left lower extremity edema and foot drop likely peroneal and posterior tibial neuropathy from compression. He was unable to ambulate.

Labs were significant for CPK levels over 100,000 U/L and rising requiring urgent hemodialysis, leukocytosis, hyponatremia, and urine toxicology positive for cocaine and opiates. Transaminitis in the thousands with hepatocellular injury patterns was noted, creatinine of 4 and blood urea nitrogen in the 40s with no baseline to compare, and mixed triple acid-base disorder and hyponatremia (Table 1).

**Table 1:** Patient's lab data during emergency room stay and hospitalization, and up to discharge. CPK: Creatinine phosphokinase; BUN: Blood urea nitrogen; ALT: Alanine transaminase; SGPT: Serum glutamic-pyruvic transaminase; AST: Aspartate aminotransferase; SGOT: Serum glutamic-oxaloacetic transaminase; ANCA: Antineutrophil cytoplasmic antibodies; HIV: Human immunodeficiency virus; HD: Hemodialysis.

Lab component	Latest reference range	Patient value on admission	Hospital-day 1	Day 3	Day 8 HD	Discharge
CPK	39 - 308 U/L	118,923 (H)	89,377 (H)	61,026 (H)	1,618 (H)	397 (H)
BUN	7 - 18 mg/dL	44	46	62	108	20
Creatinine	0.7 - 1.2 mg/Dl	4.1	4.9	7.5	12.9	1.2
ALT (SGPT)	<=41 U/L	1,239 (H)	1,129 (H)	1,102 (H)	626 (H)	18
AST (SGOT)	<=40 U/L	2,583 (H)	1,663 (H)	1,045 (H)	783 (H)	21
Cytoplasmic (C-ANCA) AB	Negative	Negative	Negative	Negative	Negative	Negative
Perinuclear (P-ANCA) AB	Negative	Negative	Negative	Negative	Negative	Negative
Atypical ANCA	Negative	Negative	Negative	Negative	Negative	Negative
HIV 1,2 AG/Ab	Non-reactive	Non-reactive	N/A	N/A	N/A	N/A

**Figure 2:**
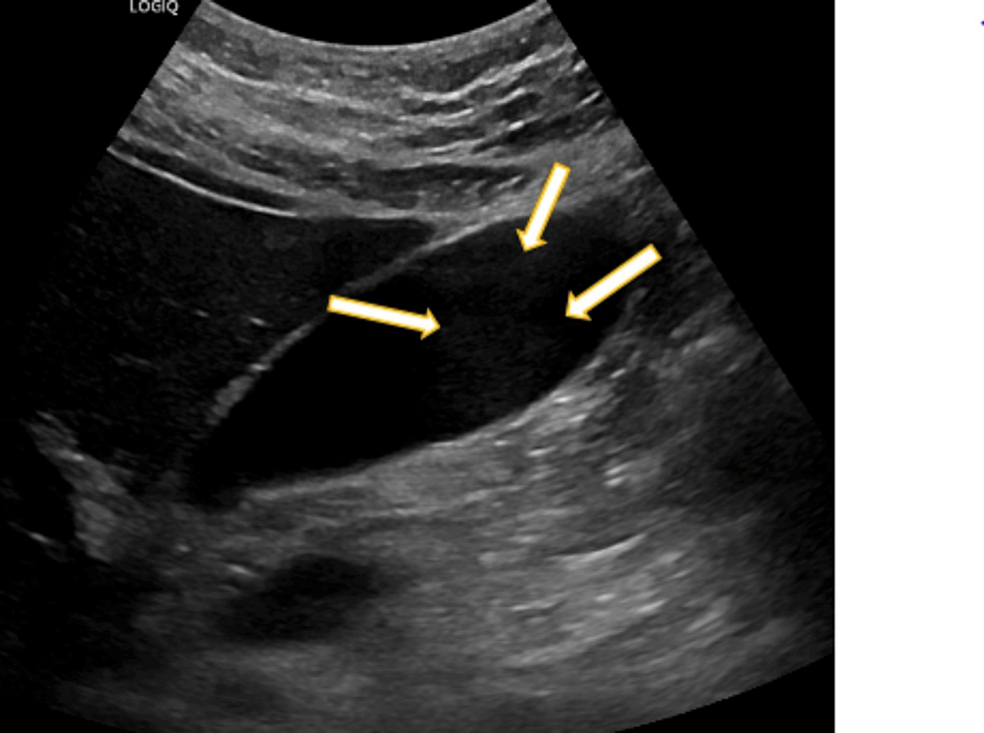
Ultrasound of liver. Sludge is present within the gallbladder fossa (yellow arrows). Views provided of the gallbladder demonstrate some low-level echoes within the gallbladder fossa with a fluid/debris level compatible with sludge.

Imaging and trauma series, including a CT chest and CXR were completed with no fractures, foreign bodies, or gas noted. Deep venous thrombolysis (DVT) was ruled out with Doppler ultrasound. Abdominal ultrasound demonstrated hepatomegaly (18.8 cm) and gallbladder sludge (Figure 2).

**Figure 3:**
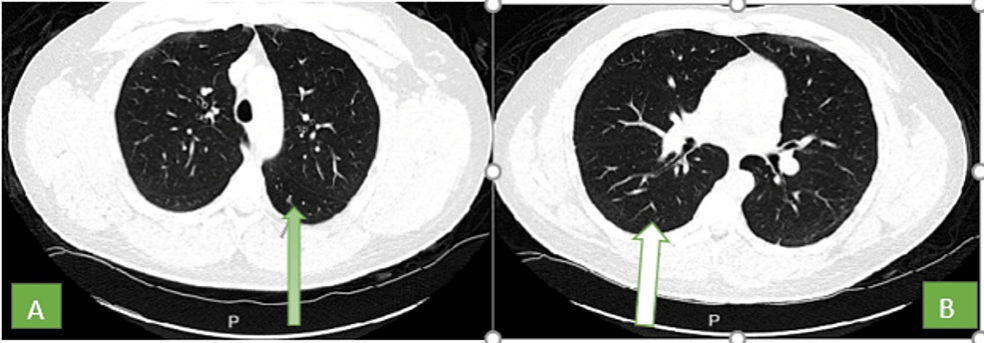
CT of the chest without IV contrast. A: Linear opacity along the dependent region of the left lower lobe; finding represents scarring, and ground glass nodule within the superior segment of the left lower lobe measuring approximately 4 mm (thick green arrow). B: There is a subpleural nodule along the posterior aspect of the right lower lobe superior segment measuring approximately 4.1 mm. There are ground glass opacities at the periphery of bilateral upper lobes anteriorly, probably interstitial lung disease.

Slightly increased echogenicity of the renal parenchyma compatible with the medical renal disease was noted. Non-contrast CT abdomen with no intra-abdominal pathology was noted. Reticular as well as ground glass opacities were seen throughout the periphery of the lungs on the CT chest suggestive of interstitial lung disease for which Pulmonology was consulted (Figure 3).

**Figure 4:**
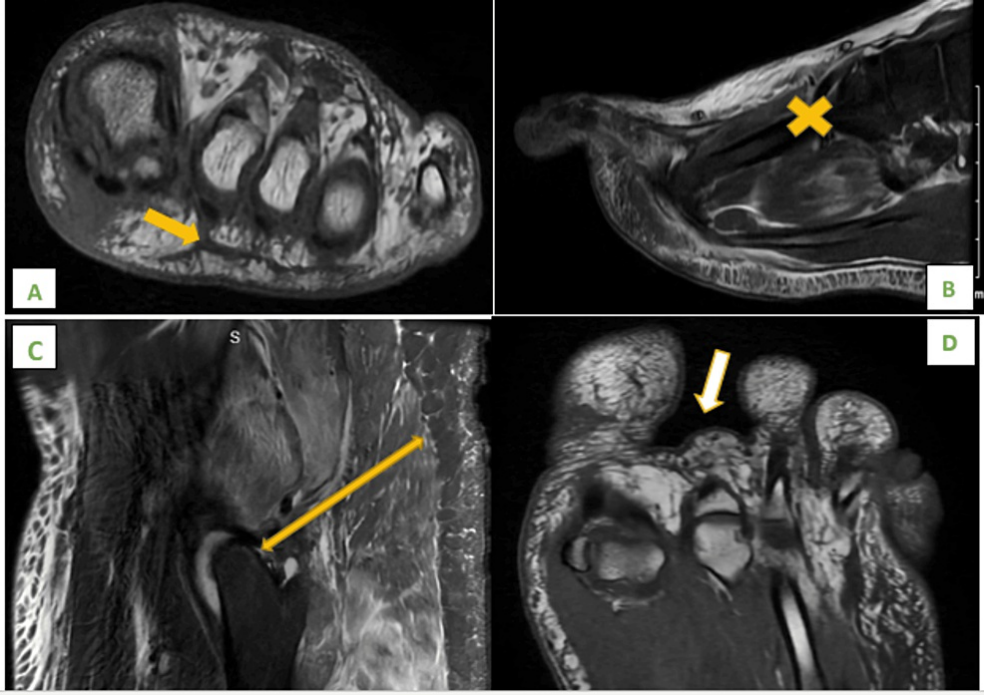
MRI of the left foot and left hip. A: Joint effusions are seen throughout the interphalangeal joints and metatarsophalangeal joints. B: Minimal first and second intermetatarsal space bursitis + small amount of fluid surrounding the plantar fascia. A large amount of soft tissue edema on the dorsal aspect of the left foot. C: Enlargement as well as edema of the left gluteus minimus, gluteus medius, gluteus maximus, quadratus femoris, abductor magnus, and piriformis muscles with edema coursing in the superficial as well as deep fascial planes of the muscles surrounding the left hip extending into the left hemipelvis due to myositis. D: Edema throughout the intrinsic muscles of the foot as well as throughout the deep and superficial fascial muscular planes.

MRI demonstrated edema throughout the intrinsic muscles of the foot as well as throughout the deep and superficial fascial muscular planes concerning for infectious, inflammatory, or post-traumatic, surgery evaluated and also ruled out necrotizing fasciitis and compartmental syndrome (Figure 4).

A 2D echocardiogram showed normal left ventricle and right ventricle systolic function with possible bicuspid aortic valve with mild aortic insufficiency; however, there were no vegetations seen. Aggressive hydration and autoimmune work-up were requested and antibiotics for cellulitis were started. Dermatology was consulted during his stay for pruritic rash (Figure 1) thought to be 2/2 to bacterial folliculitis and started on hydroxyzine for itching.

**Figure 5:**
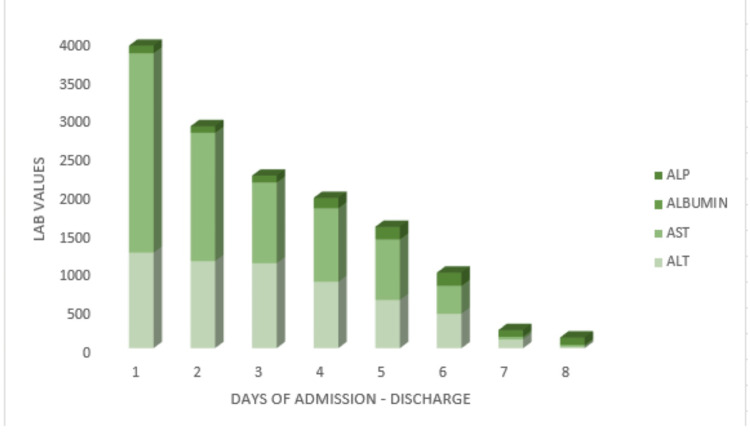
Graph demonstrating the liver enzyme patterns throughout admission until discharge. ALP: Alkaline phosphatase (U/L); ALT: Alanine transaminase (U/L); AST: Aspartate aminotransaminase (U/L).

**Figure 6:**
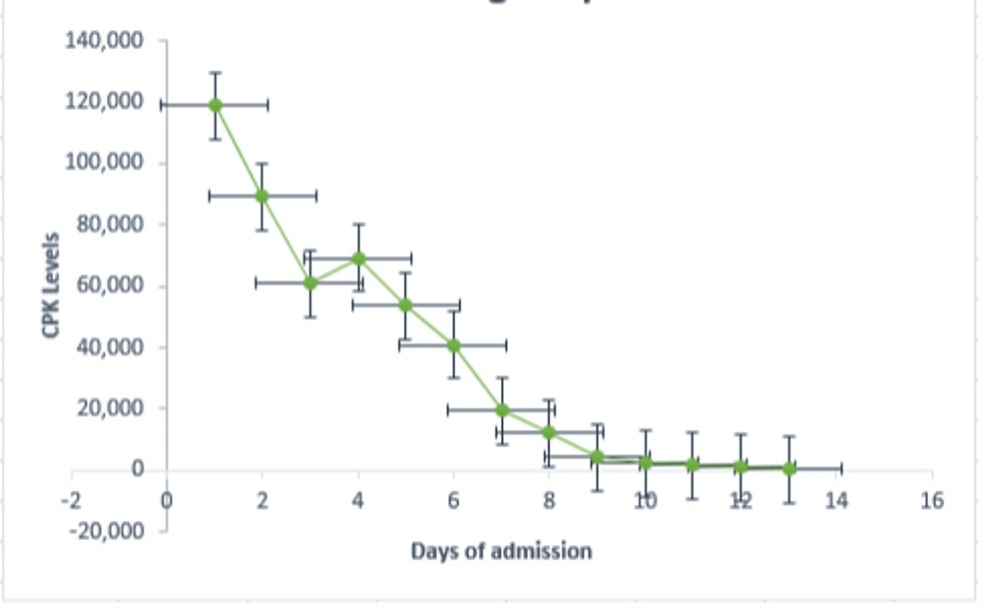
Creatinine phosphokinase level during hospitalization and at discharge. CPK: Creatinine phosphokinase (U/L).

Gastroenterology was consulted for his transaminitis, and expectant management was recommended as it was taught to be secondary to his rhabdomyolysis. Rheumatology was also consulted as he had a positive family history of a mother who died from complications from lupus. His antinuclear antibody (ANA) test was negative, and a possibility of a levamisole-induced vasculitis was made; however, ANCA and levamisole level screening were unremarkable (Table 1). The patient's condition improved and he was deemed stable for transfer to an acute inpatient rehab facility for comprehensive rehabilitation, inclusive of addiction counseling. This patient was managed by Neurology, Nephrology, Gastroenterology, Rheumatology, Pulmonology, Dermatology, and General Surgery during this hospitalization. Labs were trended following admission and starting antibiotics and fluids, He continued to improve with hemodialysis. Graphs demonstrate the trends of his lab data during hospitalization and before discharge (Figure 5, 6).

## Discussion

Multi-organ injury secondary to cocaine use is a recognized entity. Our patient had systemic injury secondary to toxic levels of CPK and rhabdomyolysis. Nephrotoxicity secondary to cocaine has been described by a multifactorial mechanism [5]. The spectrum of renal assault can be acute, chronic, or via hypertension-induced chronic kidney disease after a long use [6]. The acute insult can be via rhabdomyolysis as our patient, thrombotic microangiopathy, vasculitis, acute interstitial nephritis (AIN), and kidney infarction. Muscle breakdown further worsens the rhabdomyolysis. 

Cocaine-induced hepatocellular injury is secondary to several mechanisms; one is through the P450 pathway due to the toxic metabolite named norcocaine, and another mechanism is via ischemia due to vasoconstriction of the central hepatic vein. The injury occurs in a rapid timeline within hours, with a pattern of AST > ALT 10-12 times the upper limit of normal and can last for days. Cocaine-induced liver injury can also elevate lactate dehydrogenase (LDH) causing an ALT/LDH ratio of 0.5 which differentiates it from other etiologies of shock liver with an ALT/LDH ratio of 1-1.5. Rhabdomyolysis and fever are frequently associated with cocaine-induced liver assault, as seen in our patient. N-acetylcysteine (NAC) may be helpful; management is usually supportive [6,7]. 

**Figure 7:**
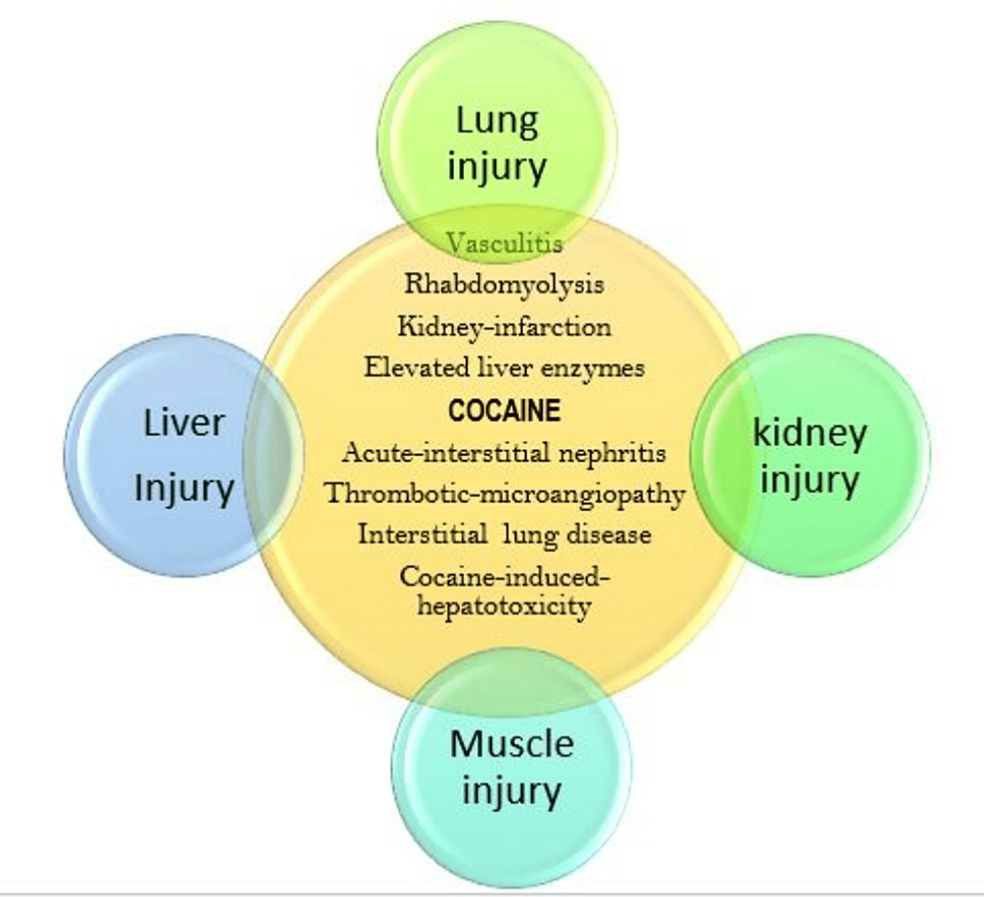
Wide-spectrum of cocaine-induced kidney, liver, lung, and muscle injury. CIH: Cocaine-induced hepatotoxicity.

Acute pulmonary toxicity resulting from smoking crack cocaine, referred to as "crack lung" is a syndrome consisting of diffuse alveolar injury and hemorrhagic alveolitis [8]. This occurs within 2 days of smoking crack cocaine. It may present as diffuse alveolar hemorrhage or as bronchoalveolar lavage eosinophilia on bronchoscopy. Pulmonary eosinophilia is also a risk factor following intranasal cocaine use, to mention a few [9]. Diffuse interstitial disease, as seen in our index patient (no noted emphysematous changes) on his chest CT scan, may benefit from transbronchial biopsy at the time of flexible bronchoscopy, if no contraindication is documented [10-12]. Figure 7 depicts the wide spectrum of cocaine-induced organ injuries. Note that vasculitis is often associated with adulterated cocaine. 

## Conclusions

Cocaine is a major cause of end-organ toxicity in almost every organ system in the body; this occurs mainly via its hemodynamic effects with its most prevalent use in North and South America, Western and Central Europe, and Oceania. The sequelae of multi-organ injuries and failure secondary to cocaine toxicity are costly and affect the young and old equally. A multidisciplinary approach is needed to effectively manage the consequences. Further reporting and research are needed to combat this pandemonium.
